# Medical Recognition Again

**Published:** 1887-11

**Authors:** 


### Editorial.

### MEDICAL RECOGNITION AGAIN.

The *Journal of the American Medical Association,* in its issue for August 6th, took occasion to review an editorial in the August number of this Journal, and in effect to accuse its editor of a lack of comprehension of the following resolution, passed by the A. M. A. at its last meeting in Chicago :

*Resolved,* That the regular graduates of such dental and oral schools and colleges as require of their students a standard of preliminary or general education and a term of professional study equal to the best class of the medical colleges of this country, and embrace in their curriculum all the fundamental branches of medicine, differing chiefly by substituting practical and clinical instruction in dental and oral medicine and surgery, in place of practical and clinical instruction in general medicine and surgery, be recognized as members of the regular profession of medicine, and eligible to membership in this Association on the same conditions and subject to the same regulations as other members.

This action we commended, and expressed the belief that if it were strictly enforced its influence upon dentistry and our dental schools would be of the greatest benefit. But we quoted from an editorial in the *Journal* the following extract, and expressed the fear that it did not indicate that this standard was to be preserved in good faith in the admission of dentists to the International Congress. We were most heartily in favor of accepting the reputable D. D. S. as a qualification for admission, but we opposed the recognition of Delavan and other like fraudulent diplomas, or the issuing of membership tickets to notorious dental quacks. We desired, too, that when the D. D. S. was recognized, it be accepted for what the Chicago resolution really pronounced it—*a degree in medicine.* The editorial in the *Journal* said :

" In regard to the registration of educated dentists, about which there has been some question, it is sufficient to say that the same rule will be followed as governed at the London Congress of 1881. The establishment of a Section of oral and dental surgery is a full admission that it constitutes a part of the domain of general medicine and surgery, and that all who, by education and proper legal authority, practice in that special department, are ' members of the regular medical profession.' At the London Congress they registered with the common prefix ' Dr.,' as did a large portion of eminent members of the profession in other departments.

"At the Congress at Washington it will be proper for them to register with the title Dr., M. D., D. M. D. or D. D. S., according to the term of the authority conferring upon them the right to practice their profession."

We said that the Chicago action "positively announced that the possession of the diploma of a school which came up to the requirements of the resolution would entitle its holder to admission to the International Medical Congress," and the *Journal* remarks, that as the resolution "not only contained no such positive announcement, but actually makes no allusion, directly or indirectly, to the International Medical Congress, it is difficult to account for such an assertion."

Let us see whether the editor of the *Journal* is dealing ingenuously, or is endeavoring by a forced construction to escape from a disagreeable dilemma. The word "positively" was a misprint for "practically," but as the correction in the proof failed to become incorporated in the text, we will even take the paragraph as it reads.

The rules of admission, as published, prescribed that "The Congress will be composed of members of the regular medical profession." The rule for the London Congress was as follows: "The Congress will be composed of medical men legally qualified to practice in their respective countries." It did not accept or acknowledge as a qualification any degree save that of a regular medical school. There was no modification of this rule, except that in regard to invited gusts. All members of "the regular profession of medicine" were entitled to membership. The resolution of the A. M. A. declared that the regular graduates of such dental and oral schools and colleges as came up to the required standard should "be recognized as members of the regular profession of medicine." If this is not an announcement that "the possession of such a diploma would entitle its holder to admission to the International Medical Congress," then there is no force in language, and if the respected editor of the *Journal* cannot so see it, we submit that he is the one who is decidedly "mixed." Besides, the editorial quoted positively asserts that it would be proper for dentists to register with the title of D. D. S., and there was no possible authority for the declaration except that of the resolution.

Again, we said "we were not certain that the authority of the American Medical Association to establish new degrees in medicine would be respected by all the world, or that its right to modify the rules of admission to a congress with which that association had really nothing to do would be acknowledged by foreign members, but it at least established a valuable precedent."

Upon this the editor of the *Journal* comments as follows: "If our readers will turn to the number of the *Journal* where the resolution and the action of the Association thereon are recorded, they will seek in vain for the slightest allusion to the establishment of any new degrees in medicine."

And yet the resolution positively declares that the possessors of the degree of Doctor of Dental Surgery, for that or its equivalent is the only degree which "dental and oral schools and colleges" confer, shall be recognized as "members of the regular profession of medicine." Before that action, no degree except that of a medical college admitted to such membership, but now it was announced that another diploma should confer the same privilege, and if this is not establishing a new degree in medicine there is no force in logic or meaning in words. When the editor of the *Journal* declared that "At the Congress in Washington it will be proper for them to register with the title Dr., M. D., D. M. D., or D. D. S.,' in a congress which is to "be composed of members of the regular medical profession" only, and almost in the same breath denies that a new degree in medicine, or of admission to the regular medical profession, has been established, we again wonder who it is that is badly "mixed."

The result of this unauthorized announcement was what we feared. It swelled the numerical strength of that meeting and added something to its funds, but at the expense of the good repute of the Dental Section, for in consequence of it some of the most notorious quacks in dentistry, men without qualification or reputation, possessed only of a bad notoriety, registered as members, thrust themselves into the front ranks on every occasion of display, and only took back seats when force was threatened for their expulsion. We are jealous for the good reputation of the profession to which we belong, and when an attempt is made to swell the ranks of any association or meeting of professional men at the expense of dentistry, by admitting in its name those whom dentists repudiate and whom every reputable practitioner shuns and refuses to acknowledge, we shall take occasion to enter our vigorous protest.

We are as anxious as any one to see dentistry properly acknowledged, and to assist in the preservation of a high standard of qualification, but as dentists we shall not add to our own self-respect, or gain the esteem of others, by allowing the disreputable to be thrust upon us. If the American Medical Association will stick to the terms of the Chicago resolution, and recognize those, and only those, who can comply with its requirements, it will assist us materially; but if all its members place upon that expression the same construction that the *Journal* seems to do, and have as little comprehension of the wants and aspirations of all progressive dentists as the editorial in question evinces, we can only say that the less they meddle with dentistry the better will it be for its reputation.

